# The effects of social networks on HIV risk behaviors among Vietnamese female sex workers: a qualitative study

**DOI:** 10.1186/s41256-024-00346-1

**Published:** 2024-01-23

**Authors:** LongHui Zhou, Yi Liu, Dan Liu, ChenChang Xiao, JiaYu Li, MengXi Zhai, Xin liu, Bin Yu, Hong Yan

**Affiliations:** 1 Department of Epidemiology and Health Statistics, School of Public Health, Wuhan University, Donghu Road, Wuhan, 430071 China; 2 Department of Psychology, Wuhan University, Wuhan, China; 3 Wuhan City College, Wuhan, China

**Keywords:** Social networking, HIV prevention, Sex Workers, Qualitative research, Asia, Risk factors

## Abstract

**Introduction:**

Female sex workers (FSWs) experience heightened vulnerability to HIV and other health harms, and cross-border FSWs face additional challenges due to language issues, higher mobility, and weaker negotiation skills. Given the critical role of social network factors in HIV transmission, it is imperative to explore the social network characteristics of Vietnamese cross-border FSWs in China to enhance AIDS prevention and control.

**Methods:**

We conducted semi-structured interviews with 22 Vietnamese FSWs in Hekou County, Yunnan Province from May to July 2018. The samples were selected using a purposive sampling strategy and stopped when reached theoretical saturation. Data collection and analysis were conducted iteratively to identify themes within the data. Participants reported their social relationships and how these relationships affected their HIV risk behaviours. All the interviews were recorded, transcribed verbatim, and reviewed. Thematic analysis was used to analyse the data.

**Results:**

Among 22 Vietnamese FSWs, the median age was 23.5. Concerning social networks, interviews revealed that their social networks were composed of three components: Workplace networks (customer, boss, colleague), Hometown networks (spouse or boyfriend, family member, fellow villager), and Social institutions networks (Chinese social institutions network, Vietnamese social institutions network). None of these networks can simply support or hinder Vietnamese FSWs’ preventive high-risk HIV behaviours, and the impact is achieved through each network’s ways. Within the workplace network, the predominant influence is the ascendancy-submissiveness dynamic that exists among customers, bosses, and VFSWs. In the hometown network, familial responsibilities emerge as the principal factor impacting VFSWs. Meanwhile, within the social institution network, pivotal roles are played by the Chinese CDC and the Vietnamese government in the dissemination of HIV knowledge.

**Conclusions:**

The social networks of Vietnamese female sex workers exert a dual impact on high-risk HIV behaviors. Interventions should be designed and tailored to address the specific contextual factors and challenges associated with social networks among cross-border FSWs in China and other similar settings.

**Supplementary Information:**

The online version contains supplementary material available at 10.1186/s41256-024-00346-1.

## Introduction

As of 2020, China has an estimated 1,053,000 extant cases of AIDS [1]. Data procured from the National Bureau of Statistics delineates a consistent augmentation in the incidence of AIDS in China from 2011 to 2018 [2]. Sexual behavior emerges as the predominant conduit of HIV transmission, with heterosexual transmission accounting for approximately 70% of cases. Notably, unsafe sexual practices, such as commercial sex, significantly contribute to this mode of transmission [3].

Owing to their profession, Female Sex Workers (FSWs) are often engaged in numerous high-risk sexual behaviors, including unprotected intercourse, multiple sexual partners, and high mobility. This makes them a vulnerable population to HIV and a “bridge population” for the transmission of HIV from high-risk groups to the general population. [4, 5]. The risk of HIV acquisition in female sex workers is observed to be elevated to a level 30 times greater than that in the broader population of adult women. [6]. Furthermore, stigma, violence, and inadequate social support pose significant barriers to preventing HIV transmission among FSWs. [7]. Cross-border FSWs, compared to local FSWs, are more susceptible to HIV transmission due to their work in foreign countries. [8]. Surveys indicate that the overall prevalence of HIV among FSWs is higher in Yunnan and Guangxi provinces on the southern border of China than in other provinces [9]. A study conducted in the China-Vietnam border area of Guangxi Province revealed an overall HIV prevalence of 0.96% among FSWs, while the average prevalence among Vietnamese Female Sex Workers (VFSWs) was 3.14% [10]. HIV prevention in cross-border FSWs presents greater challenges due to language barriers, lack of legal rights, higher mobility, weaker negotiation skills during work, among other factors [11, 12]. Therefore, it is crucial to comprehensively understand the social characteristics of cross-border FSWs and implement targeted HIV prevention measures.

HIV risk behaviors, such as condomless sex, are shaped not solely by individual factors but also by interpersonal and environmental influences. Social network factors play a pivotal role in either facilitating or impeding HIV transmission [13]. A social network is a collective of individuals interconnected through various social relationships [14], and it comprises three components: relations, structures, and functions [15]. Network relations pertain to types of relationships, encompassing partners, relatives, friends, superiors, and subordinates. Network structures include network size, type, and frequency of contact, while network functions encompass supportive and hindering effects in emotional, material, or informational terms [13, 15]. Research has demonstrated that interventions targeting social networks can curtail the incidence of HIV risk behaviors and minimize HIV transmission within intervention cohorts [16]. For instance, a randomized controlled study of social networks of Russian and Hungarian men who have sex with men revealed that the implementation of social network interventions resulted in a decrease in the rate of unsafe sex with sexual partners among participants [17]. In a qualitative study on older Chinese FSWs, different relationships in family and work networks exerted varying impacts on condom use [13].

Cross-border FSWs, due to their migrant status, exhibit distinct social network characteristics compared to local FSWs. Despite the increasing presence of Vietnamese cross-border FSWs in China [12], comprehensive information on their demographic and sociological characteristics remains scarce. Consequently, this qualitative study was designed to delve into the social network characteristics of Vietnamese cross-border FSWs in China and to comprehend how these characteristics influence their HIV risk behaviors. This understanding is pivotal for the effective prevention and control of HIV in the border regions of China.

## Methods

### Study design

Hermeneutic phenomenology aims to reveal the meaning and central structures of participants’ lived experiences, which aim to develop plausible insights to direct contact with the world [18]. This study adopted a hermeneutic phenomenology approach to figure out the social network characteristics and their impacts on HIV high-risk behavior of Vietnamese FSWs in China. Upon extensive review of pertinent literature, a comprehensive interview outline was formulated after a thorough discussion with the research team. The semi-structured interviews were designed to cover several domains, including general demographic characteristics (such as age, marital status, and education), work experience, organizational structure of sex work, HIV/STD-related conditions (including HIV knowledge, attitudes, behaviors, access to medical care, and medication), and social network characteristics (comprising of family, friends, boss, customers, etc.). The specific interview outline is provided in the supplement.

### Participants

Hekou County is located on the southeastern border of Yunnan Province, China, just across the river from Lao Cai City, Vietnam. We employed purposive sampling with the aim of encompassing a broad spectrum of survey participants in Hekou County from May to July 2018, while concurrently ensuring that our sample encapsulated Vietnamese female sex workers of varying HIV statuses, and from diverse venues and socioeconomic backgrounds. The eligibility criteria for the study were as follows: participants had to be female sex workers who self-reported providing commercial sex services in China within the preceding three months; they had to hold Vietnamese nationality; they had to be aged 16 years or older; their participation in the survey had to be voluntary; and they had to have no significant impairment in question comprehension. The study included a total of 22 Vietnamese Female Sex Workers, providing a robust and representative sample for our analysis. Ethics was reviewed and approved by the Ethics Committee of the Center for STD and AIDS Prevention and Control of the Chinese Center for Disease Control and Prevention.

### Data collection

To ensure the utmost privacy and confidentiality, personal in-depth interviews were conducted either at the local CDC (Centers for Disease Control and Prevention) or the interviewee's residence, in a serene and comfortable environment. The interview team comprised two research-trained graduate students and a local CDC outreach worker. The outreach worker, trusted for their consistent provision of condoms and knowledge dissemination services to the Vietnamese FSWs, helped us establish a rapport with them. Several trained and bilingual interpreters were recruited to help with the interview. Before the interview, written informed consent was obtained from the participants, and each interviewee was assigned a number. With verbal consent and permission granted, the entire interview process was recorded for further analysis. The interviews typically lasted between 30 and 45 min, and participants were free to withdraw from the interview at any time. Upon completion of the interview, participants would receive a compensation of 100 yuan. Throughout the interview process, the interviewers would use a memorandum to record facial expressions, environmental characteristics, and other relevant details of the interviewees. The sample size was determined based on the principle of data saturation, whereby the point at which new interviewees could no longer contribute new themes or insights was used to determine the final sample size.

### Data analysis

Following each interview, the audio recordings were transcribed verbatim, and the transcripts were reviewed to identify nonverbal cues and other contextual information that could help interpret the data. In the process of reviewing the transcripts of the audio recordings, our researchers engaged in a meticulous auditory examination of the recordings. This ensures the accurate transcription of verbal expressions, encompassing non-verbal auditory cues such as laughter, sighs, or other vocalized expressions. Any significant actions demonstrated by the interviewees during the course of the interview were promptly documented in our memorandum. This rigorous approach ensures a comprehensive understanding of the context, enhancing the richness and depth of our qualitative analysis. Any gaps or uncertainties in the transcripts were addressed by contacting the interviewees. The audio recordings, memos, and interview transcripts play a crucial role in providing a robust audit trail for the research.

An inductive thematic analysis approach was applied in the study [19, 20]. Two researchers conducted a meticulous examination of the interview transcripts, utilizing NVIVO12 software to annotate pertinent information, draft memos, and perform detailed line-by-line coding. Each researcher devised an individualized coding tree, meticulously illustrating the main and sub-themes of their coding. A research team, composed of two professors of epidemiology and a professor of social psychology with expertise in qualitative research, performed a thorough review of the identified themes. Following an in-depth discussion involving all five members, the team finalized the themes and sub-themes with precision, employing the innovative coding framework to aid further analysis. This iterative process continued until no novel themes emerged, culminating in the definitive analysis outcomes. All discrepancies among the researchers were diligently addressed, and a consensus was reached regarding the final themes.

## Results

### Sample characteristics

As delineated in Table 1, the survey encompassed 22 participants. The median age of the participants was 23.5 years. A majority of the participants (n = 19; 86.4%) originated from rural areas, while a minority (n = 3; 13.6%) hailed from urban areas. A substantial proportion (77.3%) of the sex workers disclosed having a regular sexual partner, such as a husband or boyfriend. Over half of the participants (63.6%) reported having experienced pregnancy at least once. In terms of HIV status, 2 participants (9.1%) reported being HIV positive, while 5 (22.7%) had never undergone HIV testing.

**Table 1:** Demographic characteristics of FSWs

Characteristics	N = 22
Age; median (interquartile range)	23.5(20,34)
Wages (yuan); Freq (%)	
< 5000	7(31.8%)
5000–9999	9(40.9%)
> 10,000	6(27.3%)
Registered permanent residence; Freq (%)	
Rural areas	19(86.4%)
Urban areas	3(13.6%)
Educational attainment; Freq (%)	
Primary school or lower	9(40.9%)
Junior middle school	7(31.8%)
Senior high school or higher	6(27.3%)
Regular sexual partner (husband/boyfriend); Freq (%)	
Yes	17(77.3%)
No	5(22.7%)
Ever been pregnant; Freq (%)	
Yes	14(63.6%)
No	8(36.4%)
HIV test; Freq (%)	
Positive	2(9.1%)
Negative	15(68.2%)
Never tested	5(22.7%)

### Qualitative findings

**Fig. 1:**
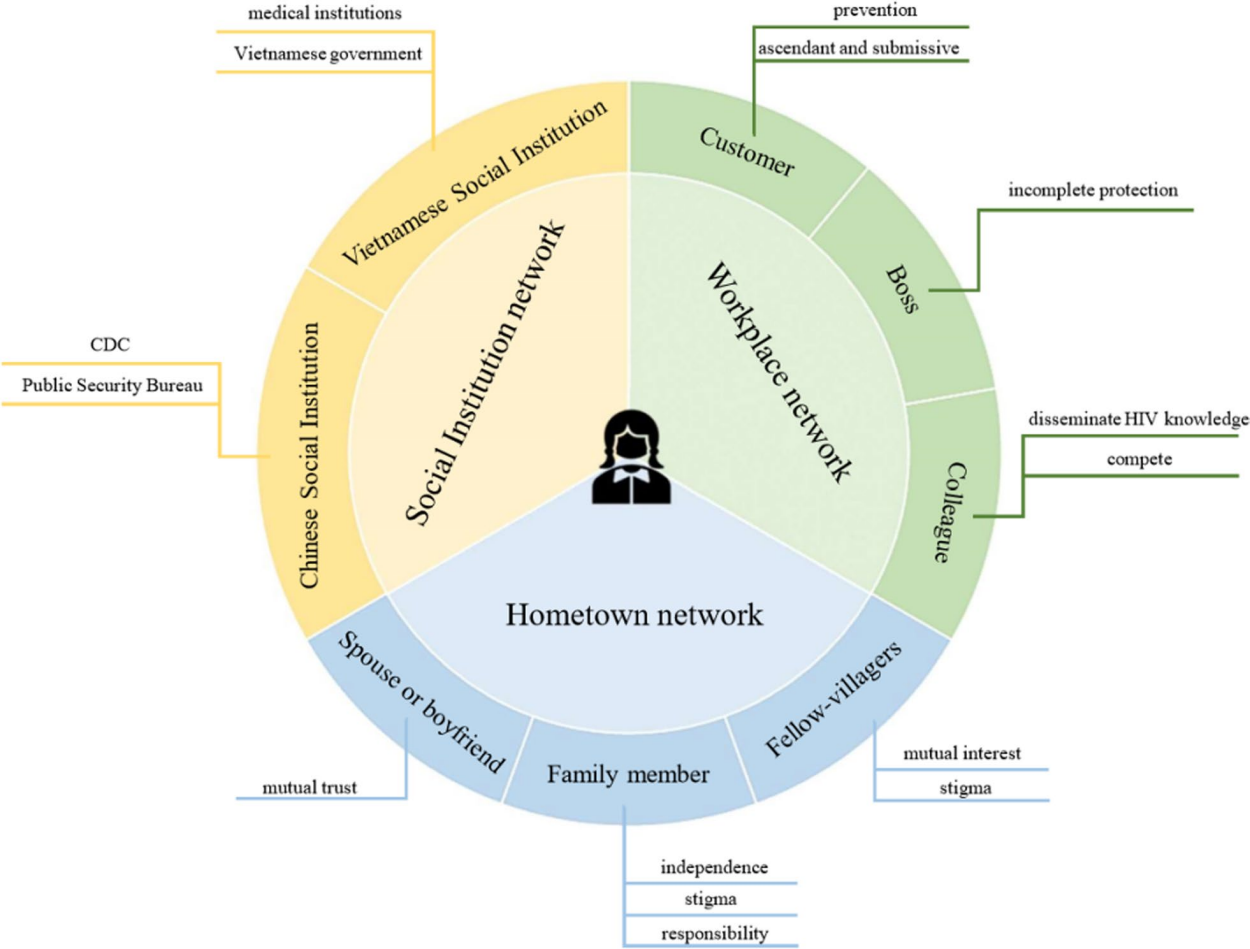
Main components of the social network of Vietnamese female sex workers

Figure 1 presents the key emergent themes and subthemes in the qualitative data. The results showed that the social networks of Vietnamese FSWs mainly involved three components: Workplace networks, Hometown networks, and Social institutions networks.

### Workplace networks

Workplace networks were composed of customer network, boss network, and colleague network. These three types of relationships could have both supportive and hindering effects on preventing high-risk HIV behaviors.

#### The ascendant position of customers leads to high-risk behaviors

The interactions between Vietnamese FSWs and their customers were typically characterized by a transactional, one-off nature. On average, Vietnamese FSWs catered to between four to six customers daily, with the majority being Chinese customers. Many Vietnamese FSWs would refuse to serve compatriots or drunk customers. This reluctance stemmed from the societal stigma attached to their profession, which discouraged them from disclosing personal information to Vietnamese clients. Besides, drunk customers posed a greater challenge to negotiating condom use and often exhibited disrespectful behavior during sexual activity, which can lead to the condom breaking easily. One Vietnamese FSW, when asked why she was reluctant to take on Vietnamese and drunk customers, replied:

*“When Vietnamese customers come in, they tend to ask a lot of questions, which I don't like to answer. That's just how it is. I don't serve drunk customers anymore because it's too tiring and sometimes they can be argumentative and cause trouble.”(VFSW 10)*

Customers occupied an ascendant position during the commercial sex act. Vietnamese FSWs often encountered communication barriers and other obstacles when negotiating with customers, which could render them feeling powerless and submissive in such transactions. This ascendaancy and submissiveness dynamic was identified as a potential barrier to the prevention of high-risk HIV behaviors. Within this context, customers frequently withheld the preparation of condoms during sexual encounters and instead made requests for additional payment in lieu of their use. Under financial pressure, Vietnamese FSWs might acquiesce to such demands, thereby exacerbating the risk of HIV transmission.

*“If the customer feels uncomfortable with using a condom during oral sex, then they may not want to continue with sexual activity.”(VFSW 01)*

*“There was one time when there were no other customers around, and the customer offered to pay extra money to have sex without a condom, so I agreed (to have sex without condoms).”(VFSW 06).*

Almost all the Vietnamese FSWs expressed a willingness to initiate condom use. However, approximately 30% of customers declined this request, and there were instances of condom removal during sexual activity or condom breakage due to rude actions. Vietnamese FSWs were often at a disadvantaged stage when negotiating with customers who refuse to comply with safe sex practices and engage in rude behavior. This dynamic increases the likelihood of Vietnamese FSWs acquiescing to customers’ requests for high-risk sexual practices, thereby making them a target group for high-risk HIV behaviors.

While some Vietnamese FSWs took proactive measures to address requests for high-risk behavior by customers, not all of these measures proved effective. For instance, some Vietnamese FSWs reported conducting visual inspections of their customers' genitals for abnormalities before engaging in oral sex. In cases where a condom is broken during sexual activity, some Vietnamese FSWs some Vietnamese FSWs were unsure of how to respond. Most would replace the condom immediately, followed by vaginal cleansing with toothpaste or cleansing agents, and the intake of contraceptives and pain medication. However, as vaginal cleansing occurred post-sex, its efficacy remained uncertain.

*“I would put a new condom back on and then wash that area after it (sex activity) was over.”(VFSW 04)*

*“Once having sex with a customer, the condom broke, and then ejaculated inside. In addition to the use of birth control pills, I did not think of using any other drugs to prevent disease.”(VFSW 06)*

The willingness of some customers to engage in high-risk sexual behavior compelled VFSWs to educate themselves about STI prevention, yet the accuracy and reliability of the knowledge couldn’t be guaranteed.

#### Bosses provide incomplete protection

The dependency of Vietnamese FSWs on their bosses was significant, as they often acted as intermediaries between the FSWs and their customers. In most cases, Vietnamese FSWs worked under one boss and were reluctant to be transferred to another location by the boss, as this meant a further reduction in wages and a longer distance from their homes. Additionally, the risk of provocation by the previous boss cannot be ignored.

*“If we want to change bosses, we have to share the earnings not only with the new boss but also with the original boss.”(VFSW 02)*

*“There is a rule here. If you change the workplace, he (the original boss) will trouble you. He will get someone to beat you up. He won't give you your money, he won't let you go to work.”(VFSW 11).*

*“I wouldn't want to go if the *boss* rented me out. I am homesick and don't want to go.”(VFSW 12)*

Vietnamese Female Sex Workers relied significantly on their bosses to facilitate their smuggling activities. At the same time, Vietnamese FSWs faced challenges in surviving independently in China due to language barriers. In the event of an arrest by the Public Security Bureau, the boss might assist in posting bail. This dependence on the boss further diminished the negotiating power of Vietnamese FSWs. The dependence increased the likelihood of acquiescence to the boss’s arrangements.

The precarious reliance of Vietnamese FSWs on their bosses was a concerning reality, underscored by the latter’s coercive role in their work. This situation was compounded by the illegal nature of sex work and the bosses' close ties with the local police, further reinforcing their control over the FSWs. Several Vietnamese FSWs stated:

*“I don’t feel like to do that (serving the *customer*). But it is the boss who solicits these customers, so no matter if they are Chinese or Vietnamese, I have to take.”(VFSW 08)*

*“One day I could not stand the pain and I did not want to go to work, but the boss forced me to go to work anyway.”(VFSW 10)*

Given the dependence and coercive role, the attitudes of bosses towards HIV-risk prevention measures were of paramount importance. However, these attitudes were often ambivalent. While safeguarding the health of Vietnamese Female Sex Workers may yield long-term benefits for bosses, excessive adherence to HIV prevention measures could potentially dissatisfy customers. Consequently, the protection provided by bosses against high-risk behaviors among Vietnamese FSWs was often incomplete.

In general, most of the bosses would provide information about HIV prevention and mandate Vietnamese FSWs to use condoms during service.

*“The boss also tells us to use condoms. We have sex with customers every day, but we don't know which one has a disease. If we get *infected*, the money we earn is not even enough to treat the disease, so we have to use condoms to protect ourselves.”(VFSW 02)*

In the pursuit of financial gain, the bosses of Vietnamese Female Sex Workers demanded the use of condoms during sexual activity, yet the condoms they provided were often of poor quality. Driven by the same profit motive, the bosses did not consistently insist on measures to prevent high-risk HIV behavior. Alarmingly, many bosses discouraged the use of condoms, particularly for FSWs engaging in sexual activity for the first time.

*“Usually people don't use condoms *when* having sex for the first time, he (the boss) said so.”(VFSW 10)*

#### Colleagues disseminate HIV knowledge and compete

The colleagues of Vietnamese FSWs constituted the most frequent and intimate contacts in their daily lives, although the number of colleagues could vary depending on the size of their workplace. Colleague networks, in general, provided valuable support for preventing high-risk HIV behaviors by facilitating the dissemination of knowledge related to sexually transmitted infections (STIs).

*“The elder colleague had told us something, that if we had oral sex without a condom, we would get some sexually transmitted diseases in our throat and mouth. I was frightened of what she said, so I wouldn’t have oral sex *without* a condom later.”(VFSW 18).*

However, competition among FSWs within the same workplace for economic gain may increase the likelihood of high-risk HIV behaviors.

*“When the customer comes and chooses me, she (the person with scars on her face) will be unhappy and wants the customer to choose her.”(VFSW 17)*

### Hometown networks

#### Mutual trust to spouse or boyfriend impedes engagement in high-risk behaviors

The majority of Vietnamese FSWs maintained relationships with husbands or boyfriends in Vietnam, typically returning home every half-month to a month, during which they engaged in sexual activity with their partners. However, due to the stigma associated with sex work, many FSWs kept their occupation and even their HIV status a secret from their partners. Several Vietnamese FSWs, when asked why they went home at intervals or whether they informed their spouses or boyfriends of their work, said:

*“I lied to him (the husband) that I am working *in* Hanoi (Vietnam).”(VFSW 10)*

*“No, I won’t tell anyone (if got AIDS). I will *remember* it alone.”(VFSW 13)*

The decision to enter the sex industry was often driven by financial constraints within the family and the husband’s inability to secure sufficient income. In some cases, husbands might even encourage their wives to engage in sex work when they were unable to financially support the family.

*“my husband was moved when he saw his sister earning money here. Then he asked me to come here and do the same job to earn money, *and* that's what happened.”(VFSW 12)*

There existed a mutual trust between Vietnamese FSWs and their spouses or boyfriends, but this trust could often be accompanied by blindness, resulting in high-risk sexual behaviors.

*“Oh, let me tell you something. If you still have this defence when you have sex with your husband in Vietnam, then it's not called a couple, it's called boyfriend and girlfriend. He will doubt that you don't trust him enough or don't love him enough.”(VFSW 05).*

Despite this, this trust could be beneficial in preventing HIV-risk behaviors, in the form of increasing proactivity and willingness to prevent contracting and transmitting HIV to their partners.

*“I would use condoms with every customer to avoid getting infected and spreading the disease back to my husband.”(VFSW 20)*

However, the high-risk behaviors of their spouses, such as unsafe sex with others and drug use, can also make this trust a source of HIV transmission.

*“My husband got infected (with HIV) by other women who would call my husband to play with them.”(VFSW 05)*

Thus, the role of spouses or boyfriends in preventing high-risk HIV behaviors among Vietnamese FSWs was dual in nature: it can be supportive, but it can also lead to HIV transmission.

#### Sense of family responsibility causes hesitation to engage in high-risk behaviors

The financial necessity to support their families or to seek independence drove Vietnamese FSWs to pursue higher profits riskily. The majority of Vietnamese FSWs came from financially challenged families, and the high earning potential of sex work had made them the primary breadwinners in many households. Moreover, FSWs with strained family relationships were more likely to seek independence.

The responsibility and obligation to raise children provided moral support for Vietnamese FSWs to engage in sex work, especially for those who were divorced or single parents.

*“I come here because of the difficulty of my family. I used to work harder but earn less in Vietnam, so I wanted to earn more money to support my children and the old.”(VFSW 02)*

However, not all FSWs chose this work voluntarily, as some family members coerced them into engaging in commercial services to alleviate financial burdens.

*“In my hometown, if the family is poor, the parents will ask the girls to come over here to do this job. It's not unusual.”(VFSW 07)*

The desire for independence or a sense of familial responsibility among Vietnamese FSWs engendered a strong need for greater financial resources. This need, in turn, could tempt them to take risks in commercial sex, thereby hindering efforts to prevent HIV-risk behavior. Although they were often the main breadwinners for their families, most Vietnamese FSWs did not want their families to be aware of their occupation. They would not disclose their HIV status even if infected, fearing that such knowledge might distress their parents. This secrecy leads to a certain level of apprehension about engaging in HIV high-risk behaviors and can be a supportive factor for HIV high-risk behavior prevention.

*“My mother will ask me (why I got the disease). She will be heartbroken. She will not understand why I have this disease, and she will be sad to know. Her health will not be good if she worried too much.”(VFSW 10)*

#### The concealment of information by fellow villagers leads to high-risk behaviors

The relationship between Vietnamese FSWs and their fellow villagers was primarily driven by reciprocal economic interests. When Vietnamese FSWs were introduced to China, an intermediary fee was often charged by their fellow villagers. In an effort to protect their vested interests, some fellow villagers withholded information pertaining to the inherent risks and potential for high-risk HIV behavior associated with sex work, instead highlighting the lucrative nature of the profession. Due to the evasion of responsibility by their fellow-villagers and the self-stigma of the Vietnamese FSWs, Vietnamese FSWs were afraid of fellow-villagers revealing their work, which resulted in the loss of contact with one another after arrival in China. The interests between fellow villagers and Vietnamese FSWs, as well as the self-stigma of Vietnamese FSWs, limited Vietnamese FSWs’ access to HIV prevention knowledge and hindered their understanding of the importance of preventing high-risk HIV behavior.

### Social institutions networks

#### Chinese institutions provide measures to prevent HIV high risk behaviors

In China, Vietnamese FSWs mainly interacted with Public Security Bureau and CDC. The Public Security Bureau’s legal mandate to investigate and apprehend individuals involved in prostitution and smuggling engenders a climate of fear and mistrust among the Vietnamese FSWs. Moreover, some bosses had some relationship with the Public Security Bureau, further amplifying the distrust of Vietnamese FSWs towards the institution and reinforcing the bosses' control over them. Conversely, the relationship between the Vietnamese FSWs and the CDC was characterized by trust and close cooperation, with the CDC being viewed as a supportive entity.

*“I didn’t mention (the fact that I was trafficked to China) because the boss has a relationship with the public security, and I don’t trust those public security officers” (VFSW 22).*

*“I don't know what to do (to prevent the disease), so I hope that I could get tested. Then I just wait for you (CDC) to come and help.”(VFSW 18:)*

This reliance on the CDC had given it a significant role in helping Vietnamese FSWs prevent high-risk HIV behaviors. Several Vietnamese FSWs stated:

*“I also listen to what you (the CDC) say and *read* the materials you send out.”(VFSW 18)*

The CDC played a pivotal role in the dissemination of HIV prevention information, provision of HIV testing services, and distribution of condoms, all of which were integral to supporting Vietnamese FSWs to prevent HIV-risk behaviors. However, concerns had been raised regarding the quality of the freely distributed condoms, which had been reported to rupture during commercial sex acts. This not only increased the likelihood of disease transmission but also posed a significant obstacle to the prevention of high-risk HIV behaviors among Vietnamese FSWs.

*“I am afraid that the condom will break easily and then I will get HIV. The quality of this condom (from the CDC) is not good.”(VFSW 15)*

#### The Vietnamese government disseminates knowledge about HIV

The Vietnamese government's efforts had been helpful for Vietnamese FSWs in HIV prevention, especially in terms of knowledge dissemination. Approximately half of the Vietnamese FSWs had reported that their understanding of HIV prevention was acquired during their academic years. The advocacy efforts of the Vietnamese government had not only heightened the awareness of some Vietnamese FSWs towards the practice of safe sex but had also enhanced their capacity to prevent HIV transmission during commercial sex encounters.

*“Well, compared to the Hekou (China), there are billboards posted everywhere in Vietnam, like staying away from drugs to not transmit HIV or *something*.”(VFSW 05)*

Vietnamese FSWs maintained strong affiliations with medical institutions and pharmacies within Vietnam. Most Vietnamese FSWs opted to return to Vietnam for treatment when confronted with health issues. However, due to the Vietnamese government's measures to manage HIV infection, one Vietnamese FSW stated that she would not return to Vietnam for testing if she was infected with HIV:

*“The hospital won’t tell the family, but it will report to the district where I live, like the village, and the governor in my village will know that I get *AIDS*.”(VFSW 20)*

## Discussion

Social networks can serve as a pivotal element of social support [21]. Conducting a social network analysis of Vietnamese Female Sex Workers can offer an all-encompassing understanding of their social support characteristics. This, in turn, can provide a reference for implementing targeted social network interventions, which are crucial for enhancing HIV prevention awareness and curbing HIV transmission among Vietnamese FSWs. This study interviewed Vietnamese FSWs to categorize their primary social networks into three distinct types: workplace networks, hometown networks, and social institutions networks. Each of these networks exerts both supportive and hindering influences on the high-risk HIV behaviors of Vietnamese FSWs.

The results of the study reveal that the central structure of the workplace network is characterized by the ascendancy of customers and the submissiveness of Vietnamese Female Sex Workers.This ascendancy-submissiveness dynamic is sustained by two primary pathways: firstly, the direct ascendable role of customers over Vietnamese FSWs; and secondly, the ascendancy of customers over Vietnamese FSWs mediated by their bosses. In the first pathway, customers, as providers of economic benefits, assert a superior status in the commercial sex act and can encounter resistance from Vietnamese FSWs through coarse and aggressive behavior. The second pathway is facilitated by the boss, who acts as an intermediary. As a beneficiary, the boss grapples with the conflicting demands of maximizing profit and ensuring the health and safety of Vietnamese FSWs. In addition to the two aforementioned pathways, colleagues of similar status to Vietnamese FSWs influence their adherence to HIV prevention measures by disseminating relevant knowledge. However, colleagues are not exempt from the competitive dynamics inherent in their work environment, which could potentially lead to risky sexual behaviors.

The dual role of bosses in workplace networks offers a promising opportunity for the implementation of social network interventions. A close collaboration with bosses can effectively mitigate health risk factors and promote occupational health among in FSWs in China [22]. Given the interconnected network among bosses in supervisory workplaces, an intervention through the Popular Opinion Leaders (POLs) model presents a promising approach [16, 23]. Interventionists can identify a group of influential bosses to act as POLs, educate them about the benefits of HIV prevention measures and healthy sexual behaviors, and leverage their social influence to disseminate this knowledge to their peers. Moreover, incentives and penalties based on changes in the incidence of sexual transmission can be provided to the bosses. [24]. The work of POLs can significantly enhance the efficacy and uptake of knowledge dissemination, which can have a substantial impact on health behavior promotion [25]. Furthermore, peer education programs for Vietnamese FSWs and customers can be conducted. Studies have shown that peer education oriented towards information support can increase condom use and self-efficacy among FSWs [26]. Additionally, for Vietnamese FSWs, the use of female condoms can serve as a proactive approach to avoid high-risk HIV behaviors and negative customer experiences [13].

In the hometown network, the influence of familial responsibilities on the decision-making processes of Vietnamese Female Sex Workers is profound. They need to protect their families from infectious diseases while upholding the traditional trust in their husbands and engaging in condomless sex. This protection to family members by Vietnamese FSWs supports FSWs’ prevention of high-risk HIV behaviors. However, their mobility makes them vulnerable to HIV transmission in both directions. Moreover, the fear of stigmatization within the hometown network places a significant mental burden on Vietnamese FSWs. They often conceal their occupation and avoid social contact [27], and this stigma also discourages Vietnamese FSWs from seeking health care in their hometowns [28].

Within the social institutions network, the Chinese CDC has earned the trust of Vietnamese FSWs. However, the crackdown on prostitution and smuggling by the Chinese Public Security Bureau, have made FSWs more obedient to their boss. In instances of ill health, stigmatized Vietnamese FSWs often hesitate to return to Vietnam for treatment due to the Vietnamese government’s HIV prevention and control policy, compelling them to stay in China and even continue their work as sex workers. Despite the Vietnamese government’s significant efforts to disseminate HIV knowledge and awareness among women, those residing in rural and impoverished areas still possess limited knowledge about HIV [29]. This is particularly concerning as Vietnamese FSWs in China tend to originate from these rural and impoverished areas.

In future research, it is imperative to regularly monitor the HIV status and mobility of cross-border Female Sex Workers [11]. The active implementation of bilingual HIV intervention programs is necessary to bolster the HIV knowledge and beliefs of Vietnamese FSWs, thereby empowering them to negotiate with customers and reducing their dependence on their bosses. For instance, an intervention program in the border area effectively increased the rate of condom use [30]. Moreover, educating Vietnamese FSWs about Pre-Exposure Prophylaxis (PrEP) is an effective way to reduce HIV incidence [31]. Furthermore, collaborative efforts with the Vietnamese government to actively implement comprehensive interventions, such as regular STD screening and cross-border referral for antiviral treatment, will also contribute to reducing HIV transmission in border areas [32]. For CDC, providing higher quality condoms, intensifying publicity and education, reducing stigma against Vietnamese FSWs, and providing them with legal avenues to pursue healthcare are effective measures to improve the prevention of high-risk HIV behaviors among Vietnamese FSWs.

This study has several limitations that should be acknowledged: 1. The findings, being derived from a qualitative study, are reliant on the self-disclosures of the participants. These disclosures may be subject to influences such as social desirability bias and other external factors; 2. The necessity of a third-party translator for the interviews could potentially lead to loss of information or inaccuracies; 3. The study qualitatively examines the influence of social networks on high-risk HIV behaviors, which may limit the comprehensiveness of the findings. 4. The involvement of CDC outreach personnel may influence the perceptions of Vietnamese FSWs towards the measures adopted by local CDC. 5. In this study, the reliance on interview data collected over five years ago does introduce certain temporal limitations. Given that cross-border female sex workers, including VFSWs, share commonalities in their positioning within social networks and the challenges they encounter, the findings of this study bear a degree of generalizability and universality. The data presented herein elucidate the influence of social networks on the HIV risk behaviors of female sex workers. These insights enhance our understanding of the relationship between social networks and HIV risk behaviors, thereby informing the tailoring of pertinent interventions to the prevailing context. 6. Our study lacks subgroup analysis. For instance, disparities may exist between subgroups of female sex workers distinguished by their HIV test results. These variations could potentially impact their HIV risk behaviors and the implementation of preventative strategies. Individuals who have undergone HIV testing may be more likely to receive guidance from healthcare providers or support groups, thus facilitating the adoption of enhanced preventative measures. In contrast, those who have not been tested for HIV may lack such support and information, thereby increasing their propensity for high-risk behaviors. Future research should complement these findings with quantitative studies that describe the size of social networks and their associations and conduct subgroup analysis.

## Conclusions

Our qualitative inquiry sheds light on the influence of social networks on HIV risk behaviors among Vietnamese FSWs, encompassing workplace, hometown, and social institutions networks. These networks can simultaneously provide support and pose obstacles in the prevention of high-risk HIV behaviors, underscoring the necessity for targeted and integrated interventions to effectively address HIV high-risk sexual behavior among Vietnamese FSWs. To effectively mitigate HIV transmission among cross-border FSWs, it is imperative that interventions are designed and tailored to address the unique contextual factors and challenges associated with social networks in analogous settings.

### Supplementary Information

**Additional file 1:** Interview outline.

## Data Availability

Research data are not shared.

## Abbreviations

AIDS: Acquired immunodeficiency syndrome
CDC: Centers for disease control and prevention
FSWs: Female sex workers
HIV: Human immunodeficiency virus
VFSW: Vietnamese female sex worker
