# Supplementary material for: The effects of social networks on HIV risk behaviors among Vietnamese female sex workers: a qualitative study
Source: Glob Health Res Policy. 2024 Jan 23;9:6. doi: 10.1186/s41256-024-00346-1 (PMC10804538; doi:10.1186/s41256-024-00346-1)
Supplement: Supplementary file 1 — Additional file 1: Interview outline. [file 41256_2024_346_MOESM1_ESM.docx]

**Interview Outline**

**（interviewees: Vietnamese female sex workers）**

Hello! Thank you for participating in today's interview. Your willingness to share your experiences is greatly appreciated. This interview aims to gather insights about your work, and your honest responses will contribute significantly to the well-being of both female sex workers and the broader community.

Throughout the interview, if you have any questions or concerns, please don't hesitate to raise them. The interview is expected to take approximately 60 minutes, and we will be recording it for the sake of accuracy. Your answers to each question will remain strictly confidential, and only the investigators will have access to the recordings, which will be used solely for academic research purposes. You can think of this interview as a casual conversation.

| **Basic information** | |
| --- | --- |
| **A101. Demographic characteristics** | |
| ①**Marital Status and Family Background** | Current Marital Status (Unmarried, First Marriage, Remarried, Divorced, Widowed, Cohabiting)? Are you currently living with your husband? Could you please specify the date (year and month, for those who have been married before) of your first marriage? Number of Biological Children? What is the gender of your children? How old are your children? What are your children currently doing (e.g., attending school, working)? |
| ②**Household Economic Status** | How would you describe your current household's economic situation? |
| ③**Personal Income and Expenditure** | Monthly Income and Income Fluctuations? What is your average monthly income from sex work (yuan)? Do you have any additional sources of income apart from sex work? If yes, where do these additional income sources come from? How much is your monthly income from additional sources (yuan)? |
| **A102.** **first sexual intercourse** | |
| ①**Timing** | Can you recall and provide the date of your first sexual experience? |
| ②**Partner** | who was involved in this first sexual intercourse? |
| ③**Voluntariness** | Were you a willing participant in this first sexual intercourse? Was there any form of coercion, deception, or pressure involved in this encounter? |
| **Work** | |
| **B201.** **Procedure of work** | |
| ①**Entry into Sex Work** | How did you initially enter the field of sex work? Was it through the guidance of someone else, or did you explore this path on your own? If someone introduced you to sex work, who was it? Can you describe how this introduction took place? What information or details were shared during this introduction process? |
| ②**Motivation** | What were the primary reasons that drove you to engage in sex work? Were there any other reasons for your choice? Did you have any reservations or hesitations before entering sex work? If so, what were your concerns or hesitations? |
| ③**Voluntariness** | Was your initial entry into sex work a voluntary decision on your part?  Did anyone use persuasion, deception, or coercion to influence your decision to enter sex work? If so, who were these individuals (e.g., friends, acquaintances, family members), and how did they attempt to influence or pressure you? What were their reasons for doing so? |
| ④**Presence and Role of Colleagues** | Did you enter sex work alone, or were you accompanied by friends or others? How did your friends or colleagues react to your involvement in sex work, and did they play any specific roles in this context? |
| **B202.Work experience** | |
| ①**Cumulative Time in Sex Work** | How long have you been engaged in sex work? |
| ②**Challenges Faced in Sex Work and Coping Strategies** | In the course of your sex work, what challenges or distressing situations have you encountered and how have you typically handled them? These could include interactions with law enforcement, issues with clients refusing payment, incidents of robbery, verbal abuse, physical violence, sexual violence, family-related challenges, interactions with other sex workers, risks of sexually transmitted diseases (STDs), and HIV. |
| ③**Future Career Plans and Reasons** | Do you plan to continue working in the sex industry? If so, what are the reasons motivating this decision? |
| **Sex Industry Organizational Structure** | |
| **C301.Workplace’s situation** | |
| ①**Number and Types of Venues** | Are you primarily working independently or within venues? How many venues are you currently engaged with? Could you specify the types of venues you work in, such as hotels, nightclubs, dance halls, KTV bars, regular bars, beauty salons, hair salons, massage parlors, saunas, street corners, roadside shops, or public squares? How long have you worked at each of these venues? |
| **C302.Work organization** | |
| ①**Boss** | What roles does the boss typically play? Which aspects of your work are managed or overseen by the boss? How do you comply with this management, and are there specific rules or regulations that you need to follow? What are the consequences for not adhering to these rules? |
| ②**Sourcing Clients and Solicitation Methods** | How do you solicit your clients? How do clients and sex workers connect in your workplace? Is it a matter of client choice, or is it the responsibility of the boss to make the arrangements? |
| ③**Working Hours and Influencing Factors** | How many days do you typically work in a month, and how many days per week? Are there specific factors that influence your work schedule? |
| ④**Average Workload and Influencing Factors** | On average, how many clients do you serve in a month? Are there specific factors that influence the number of clients you encounter, such as interactions with law enforcement or the location where you work? |
| ⑤**Location of Sexual Transactions and Decision-Making** | Where do you typically engage in sexual transactions? Who is responsible for deciding the location where sexual transactions occur? |
| **C303.** **Specifics of Sex Work** | |
| ①**Condom Use and Influencing Factors** | How frequently do you use condoms (never, rarely, often, always)? Out of 10 encounters, how many times do you not use a condom? Who typically suggests using condoms during sexual encounters? What are the reasons for using condoms or what are the reasons for not using condoms? |
| ②**Sources of Condoms** | How do you typically obtain condoms? |
| ③**Issues and Responses in Commercial Sex Activity** | What potential issues have you encountered during commercial sex encounters (e.g., condom breakage or slippage, clients removing condoms during sex, clients refusing condom use)? How often do these issues arise, and how do you typically handle them? |
| ④**Attitude Toward Condom Use** | What is your attitude towards using condoms in commercial sex activity? |
| **C304.Clients situation** | |
| ①**Demographics** | What is the nationality of your clients? Are they primarily local or from different regions? Can you provide information about the age, economic status, marital status, and reasons for seeking sexual services among your clients? |
| ②**Other Expenditures** | Do clients engage in activities beyond sexual encounters (e.g., taking you out for meals)? If so, is this initiated by the client or yourself? |
| ③**Select Clients:** | How do your select clients? Are there specific types of clients that you would refuse to engage with? |
| ④**Client Attitudes Toward Condom Use** | What are the attitudes of clients toward HIV and other sexually transmitted diseases, as well as their attitudes regarding condom use? |
| ⑤**Differences in Condom Use Among Different Clients** | Do you have regular clients? Can you describe the characteristics of these regular clients? Do regular clients exhibit different condom use patterns compared to one-time clients, and if so, why? |
| **Behavior characteristics** | |
| **D401.** **Condom Use in Regular Sexual Partnerships** | |
| ①R**egular Sexual Partners** | Have you ever had a regular sexual partner or partners in the past? How many regular sexual partners have you had from the past until now? Do you currently have a regular sexual partner (if not, please provide information about your most recent regular sexual partner)? How many regular sexual partners do you currently have? |
| ②**Frequency and Types of Sexual Activity** | How often do you engage in sexual activity with your regular sexual partner(s)? What are the types of sexual activities you engage in with your regular sexual partner(s) (e.g., oral sex, vaginal intercourse, anal intercourse)? |
| ③**Attitudes Toward Condom Use in Regular Sexual Partnerships** | What is your attitude towards using condoms in sexual activity with your regular sexual partner(s)? What are the attitudes of your regular sexual partner(s) towards HIV and other sexually transmitted diseases, as well as their attitudes regarding condom use? |
| ④**Condom Use and Influencing Factors** | Do you use condoms in sexual activity with your regular sexual partner(s) (never, rarely, often, always)? Do condom use patterns vary based on the type of sexual activity? What are the reasons for using condoms or not using condoms? |
| **D402.Condom Use and Influencing Factors (Repeated Inquiry)** | |
| ①**Changes in Condom Use Since the Onset of Sexual Activity** | Since your initial sexual activity, have there been any changes in your attitude and behavior regarding condom use? What factors contributed to these changes in your attitude and behavior concerning condom use? |
| **HIV and STD-related situations** | |
| **E501.HIV/STD-Related Knowledge, Attitudes, and Behavior** | |
| ①**Knowledge of HIV/STDs** | Have you heard of sexually transmitted diseases and HIV/AIDS? What do you know about them? How are these diseases transmitted, and how can they be prevented? |
| ②**Sources of Knowledge** | How did you acquire your knowledge about sexually transmitted diseases and HIV/AIDS? |
| ③**Self-Risk Assessment and Preventive Measures** | In your opinion, how great is the risk of you contracting a sexually transmitted disease or HIV/AIDS? What factors contribute to this risk perception? Have you taken any measures to avoid infection, and if so, what measures have you implemented? |
| ④**Consultation and Disclosure** | If you suspect that you may have infected a sexually transmitted disease or the HIV virus but are unsure, what steps would you take, and who would you consult? If you were aware of your infection with a sexually transmitted disease or HIV, would you disclose this information to others? If so, to whom would you disclose it? |
| **E502.** **HIV/STD Infection, Medical Care, and Medication Adherence** | |
| ①**Past Infections, Treatment, and Outcomes** | Have you ever been infected with a sexually transmitted disease? If so, which disease(s) did you contract, when, and how? What measures did you take? How did the treatment progress, and what is your current condition? |
| ②**Close people's Infections and Corresponding Measures** | Do you know of anyone in your social network who has been infected with a sexually transmitted disease or HIV? If so, which disease(s) did they contract, and what measures did they take? Have they consistently followed the prescribed treatment? |
| **Relationships/social networks** | |
| **F601.** **Daily Social Network** | |
| ①**Daily Contacts and Interactions** | Who do you interact with most frequently in your daily life? Do these individuals know about your current occupation, and if so, how do they feel about it? |
| ②**Issues Encountered in Work and Life, and Sources of Support** | What difficulties or problems do you often encounter in your work and life (e.g., feeling down, financial issues, illness, disputes like clients not paying, encounters with law enforcement, violence, etc.)? When you face these difficulties, are there people who can provide support? Who are these individuals, and how do they typically help you? |
| **F602.Boss** | |
| ①**Relationship with Boss** | How did you come to know your boss? What is your relationship with them? What are your feelings or thoughts about your boss? How do they influence your life and work? |
| ②**Boss's Attitudes Towards Condom Use** | Do you have discussions with your boss about knowledge of sexually transmitted diseases, HIV, and condom use? If so, what topics are discussed? What are your boss' opinions on sexually transmitted diseases, HIV, and condom use? |
| **F603.** **Colleagues** | |
| ①**Relationship with Colleagues** | How did you come to know your colleagues? What roles do they play in your life and work? |
| ②**Colleagues' Attitudes Towards Condom Use** | Do you have discussions with your colleagues about knowledge of sexually transmitted diseases, HIV, and condom use? If so, what topics are discussed? What are your colleagues' opinions on condom use? |
| **F604.** **Other People** | |
| ①**Relationship with Other People** | Do you discuss knowledge of sexually transmitted diseases, HIV, and condom use with people other than your colleagues or boss, such as outreach worker or police? Who are these individuals, and what is your relationship with them? How do they impact your life and work? |
| ②**Other People's Attitudes Towards Condom Use** | Do you have discussions with them about knowledge of sexually transmitted diseases, HIV, and condom use? If so, what topics are discussed? What are their attitudes toward your use of condoms during commercial sexual activities? |
